# An Extensive Comparison of the Effect of Anthelmintic Classes on Diverse Nematodes

**DOI:** 10.1371/journal.pone.0070702

**Published:** 2013-07-15

**Authors:** Yan Hu, Brian L. Ellis, Ying Y. Yiu, Melanie M. Miller, Joseph F. Urban, Linda Z. Shi, Raffi V. Aroian

**Affiliations:** 1 Section of Cell and Developmental Biology, Division of Biological Sciences, University of California, San Diego, La Jolla, California, United States of America; 2 United States Department of Agriculture, Agriculture Research Service, Beltsville Human Nutrition Research Center, Diet, Genomics and Immunology Laboratory, Beltsville, Maryland, United States of America; 3 Institute of Engineering in Medicine, University of California, San Diego, La Jolla, California, United States of America; Griffith University, Australia

## Abstract

Soil-transmitted helminths are parasitic nematodes that inhabit the human intestine. These parasites, which include two hookworm species, *Ancylostoma* *duodenale* and *Necator americanus*, the whipworm *Trichuris* *trichiura* , and the large roundworm *Ascaris* *lumbricoides* , infect upwards of two billion people and are a major cause of disease burden in children and pregnant women. The challenge with treating these diseases is that poverty, safety, and inefficient public health policy have marginalized drug development and distribution to control infection in humans. Anthelmintics (anti-worm drugs) have historically been developed and tested for treatment of non-human parasitic nematodes that infect livestock and companion animals. Here we systematically compare the *in vitro* efficacy of all major anthelmintic classes currently used in human therapy (benzimidazoles, nicotinic acetylcholine receptor agonists, macrocyclic lactones, nitazoxanide) against species closely related to human parasitic nematodes-*Ancylostoma ceylanicum*, *Trichuris* *muris* , and *Ascaris* *suum* --- as well as a rodent parasitic nematode used in veterinary drug discovery, *Heligmosomoides* *bakeri* , and the free-living nematode *Caenorhabditis elegans*. Extensive *in vitro* data is complemented with single-dose *in vivo* data in three rodent models of parasitic diseases. We find that the effects of the drugs *in vitro* and *in vivo* can vary greatly among these nematode species, e.g., the efficacy of albendazole is strong on *A. ceylanicum* but weak on *H* *. bakeri* . Nonetheless, certain commonalities of the *in vitro* effects of the drugs can be seen, e.g., nitazoxanide consistently shows an all-or-nothing response. Our *in vitro* data suggest that further optimization of the clinical efficacy of some of these anthelmintics could be achieved by altering the treatment routine and/or dosing. Most importantly, our *in vitro* and *in vivo* data indicate that the hookworm *A. ceylanicum* is a particularly sensitive and useful model for anthelmintic studies and should be incorporated early on in drug screens for broad-spectrum human soil-transmitted helminth therapies.

## Introduction

Soil-transmitted helminths (STHs), which include two hookworm species, *Ancylostoma* *duodenale* and *Necator americanus*, the whipworm *Trichuris* *trichiura* , and the large roundworm *Ascaris* *lumbricoides* , infect upwards to two billion people and are a leading source of disease burden in more than 400,000,000 children [1–4]. Long-term infection in children is associated with growth stunting, cognitive defects, immune defects, reduced earning, and lower school attendance. Hence, the World Health Organization (WHO) and world leaders are calling for the elimination of these parasites in children through mass drug administration as the only proven method for control. Pregnant women are also considered particularly at risk for STH disease.

Four drugs are currently approved anthelmintics for treating STHs by the WHO [5,6]. The drugs of choice are the benzimidazoles, mebendazole and albendazole (ALB); the two alternatives are the nicotinic acetylcholine receptor (nAChR) agonists pyrantel (PYR) and levamisole. All of these drugs were developed for treating veterinary parasites [7,8]. Under single-dose mass drug administration (MDA) treatment regimens for STHs, all work well against *Ascaris* , poorly against whipworms, and, with the exception of ALB, poorly against hookworms. Even in the case of ALB, which is the drug of choice for mass drug administration, examples of resistance or low efficacy against STHs have been reported [9–11]. Ivermectin (IVM), a macrocyclic lactone discovered for veterinary application [12] and used to treat filarial nematode infections in humans, is not typically used for STHs because of its relatively low efficacy against hookworms and whipworms [6]. An alternative drug uncommonly used for STHs is nitazoxanide (NTZ), which also was not originally developed to treat human STHs [7]. NTZ is typically used as six doses over a period of three days to enhance efficacy [13].

As indicated above, treatment of STHs is unique in human chemotherapy—none of the approved drugs were initially developed for use in humans or for use against human parasites. Screens for STH anthelmintics use intestinal nematode parasites like those in the genus *Heligmosomoides*, *Haemonchus* or *Trichostrongylus* [12,14–16], none of which are human parasites (for reviews of the veterinary anthelmintic literature, see 17,18). Human STHs are not incorporated into drug screening for veterinary parasites although these drugs are the source for all current human STH therapies. This leaves open the question about the utility of using human parasitic nematodes in screening for anthelmintics.

Studies on the utility of human STHs (or close relatives of humans STHs) for drug studies are very limited. Most focus on assays for hookworms [19–23] and a few on whipworms [24,25]. Studies incorporating *Ascaris* are rare, and only one study has looked at the effect of a single drug (not approved for human therapy) on all three major STHs [26]. To date there has been no single study simultaneously evaluating the effects of all major classes of anthelmintics on STHs in order to ascertain the relative strengths of using human STHs in drug screening. Neither has there been a study to comprehensively compare anthelmintic responses of major human STHs to other nematodes that do not parasitize humans. Such a study would provide a means to evaluate how well other nematodes might predict effects on human STHs and might provide some valuable information for clinical application of these drugs. To address these deficiencies and lay important groundwork for incorporating human STHs into anthelmintic drug screening, we present the first comprehensive comparison of the effects of all major classes of anthelmintics on major STHs of humans (or related species) and on two nematodes that do not parasitize humans.

## Materials and Methods

### Ethics statement

All animal experiment was carried out under protocols approved by either the UCSD or United States Department of Agriculture (USDA) Institutional Animal Care and Use Committees (IACUC). All housing and care of laboratory animals used in this study conform to the NIH Guide for the Care and Use of Laboratory Animals in Research (see 18-F22) and all requirements and all regulations issued by the USDA, including regulations implementing the Animal Welfare Act (P.L. 89-544) as amended (see 18-F23).

### Reagents and drugs

RPMI 1640 (catalog no. 11835-030), Hanks’ Balanced Salt Solution (HBSS) (pH 7.2) (catalog no. 14025-092), Fetal Bovine Serum (catalog no. 10437-028), Penicillin-Streptomycin (catalog no. 15070-063) and Fungizone Antimycotic (catalog no. 15290-018) were all purchased from Gibco. Albendazole (catalog no. A4673), ivermectin (catalog no. I8898), pyrantel tartrate, (catalog no. P7674; chosen over the other nAChR agonist, levamisole, because levamisole has toxic side effects that has resulted in it being withdrawn for use in human health in a number of places, including the United States and Canada [27]), and dimethyl sulfoxide (DMSO) (catalog no. D8418) were purchased from Sigma-Aldrich. Nitazoxanide was kindly provided by the Institute for One World Health. The new veterinary anthelmintic, monepantel, was not included in the study because it has low efficacy against many nematodes in the same genus as those that infect humans, including *Ascaris* and *Trichuris* , and thus does not qualify as a drug candidate for development and use against human STHs [26]. Drugs (10 mg) were freshly dissolved in 20 µL 100% DMSO and then diluted with 180 µl water (50 mg/mL in 10% DMSO). Ten-fold dilutions were then made in 10% DMSO. In the wells with nematodes, the drug in DMSO was diluted another 50 fold (final concentration of DMSO 0.2% in all wells). In all cases, 1/50 the final volume of the well was added as drug. In each experiment for each parasite, a no-drug control (final concentration of DMSO 0.2%) was set up in duplicate.

### Nematode maintenance

*Ancylostoma ceylanicum* hookworms were maintained in golden Syrian hamsters [28], *Heligmosomoides* *bakeri* was maintained in Swiss Webster mice [29], and *Ascaris* *suum* was maintained [30] in a four-way crossbred composite BX line of pigs. For *Trichuris* *muris* , 5-6 week old male and female STAT6-/- mice were infected *per os* with 400 infective eggs. To maintain the life cycle, *T* *. muris* adults were harvested from the cecum and large intestine at 35-40 days post infection (P.I.). Adults parasites (~10 males and ~10 females) were placed in 5 mL RPMI 1640 supplemented with 25 mM HEPES (pH 7.2, final concentration), antibiotics (100 U/mL penicillin, 100 µg/mL streptomycin; 1 µg/mL fungazole) in a 6-well plate. Plates were incubated in a 5% CO_2_ incubator at 37°C. Eggs were harvested daily and medium replaced daily for three days. Eggs were washed three times with sterile Arrowhead water and placed in 100 mm Petri Dishes and kept at room temperature in the dark for six weeks. Embryonated eggs were collected, washed in sterile water with antibiotics, and stored at 4°C for up to six months. These eggs were used for infection as needed. *Caenorhabditis elegans* wild-type strain N2 Bristol was maintained according to standard procedure [31].

### In vitro assays

Adult-staged parasites (*A. ceylanicum*, *T* *. muris* , *H* *. bakeri* ) were used where possible and practical because this stage is the target for chemotherapy in chronically infected humans. The exception was *A* *. suum* , in which intestinal parasitic fourth-stage larval (not adult) nematodes (L4) were used for practical reasons. The required number of adult-staged nematodes for this comprehensive *in vitro* study could not be readily obtained for *A* *. suum* because of inconsistent adult worm development in experimentally inoculated pigs.

Adult *A. ceylanicum in vitro* assays were carried out as described [28] with the exceptions that there were only five worms per well (separated by gender), and that the assays were allowed to go for seven days and scored on a daily basis.

*Ascaris* *suum* L4 were isolated 14 days post-inoculation (P.I.) from the small intestine of infected pigs as described [30]. The medium used for these assays was RPMI 1640 plus 5% fetal bovine serum plus antibiotics (100 U/mL penicillin, 100 µg/mL streptomycin; 0.25 µg/mL fungazole). Assays were carried out in 500 µl volume in a 24-well plate. There were five larvae per well of mixed genders because of the difficulty to discern gender at this stage of development. We note that a very different *in vitro* approach that involved following larval development and with two of the drugs used here, ALB and IVM, has previously been reported with this parasite [32].

Adult *T* *. muris* *in vitro* assays were carried out using five adults per well (separated by gender) harvested between days 35-40 P.I. and placed in the same culture conditions as used for parasite maintenance above except that the volume was 2.0 mL in 12-well plates. Adult *H* *. bakeri* were harvested from infected mice between days 18-20 P.I. and placed in the same medium used for *T* *. muris* in a volume of 500 µl in 24-well plates (five adults/well separated by gender). For all parasites, plates were put at 37°C in a 5% CO_2_ in air incubator.

For all parasites, all drugs, and all concentrations, experiments were set up with two wells per trial (separated by male and female where appropriate) and then repeated for a total of three independent trials (total of six wells per drug per concentration per parasite). Significant differences in how males or females responded to any of the drugs were never observed for *A. ceylanicum*, *T* *. muris* , and *H* *. bakeri* .

Experiments were scored as follows. Each day the motility of each parasitic nematode in each well was scored on a scale from 3-to-0: 3 represents a parasite with vigorous movement similar to control no drug; 2 represents a parasite with whole-body movements (seen without external stimulus) significantly slower than control no drug; 1 represents a parasite that was not moving on its own but moved when touched with a probe (tested at three different body locations); and 0 represents a worm that did not move even when prodded. Data shown in the figures are the result of combining all the data from all replicates and treating them as one experiment. Scoring in the range of 1-to-3 was combined as “live” and worms that had a score of 0 were treated as dead.

For *C. elegans*, ~five synchronized 44 hr L4 were transferred into triplicate wells of 24-well plates containing 500 µL special *S* *medium* and OP 50 [31] plus drug (1/50 dilution) and 8 mM 5-fluorodeoxyuridine to prevent the development of progeny. The plates were wrapped in a wet paper towel and kept in a 25 °C incubator. *C. elegans* were scored under dissecting scope on a daily basis as alive (motile) or dead (non-motile when prodded three times). The 3-to-0 motility score was not assessed for *C. elegans*, and three independent trials were carried out.

### *In vivo studies*

#### *Ancylostoma ceylanicum*

Male hamsters were infected using standard protocols [28]. On day 15 P.I., a fecal sample was collected from each hamster. The number of eggs present was counted using the modified McMaster technique [29] to ensure that the hamsters in each cage had roughly equivalent infection levels. On day 16 P.I. hamsters were weighed individually and given 0.4 mL of the relevant treatment *per os* (6 mg/kg body weight (BW) anthelmintics in sterile double-distilled water or water-only control; seven hamsters per group) through a blunt-ended gavage needle. The hamsters were sacrificed on day 21 P.I., and parasite burdens determined [28].

#### *Trichuris* *muris*

STAT6-/- mice (mixed gender) were infected with 400 infective eggs. Feces were collected from individual mice on day 39 P.I. and mice assigned to groups to ensure equivalent levels of infection amongst different treatments. The mice (six/group) were weighed individually and treated on day 40 P.I. like hamsters infected with *A. ceylanicum* except using a volume of 200 µL. Mice were sacrificed on day 45 P.I., and worm burdens were ascertained from the cecum and large intestines. No significant differences in parasite burden were seen between mouse genders.

#### *Heligmosomoides* *bakeri*

Female Swiss Webster mice were infected as described [29] and fecal egg counts were determined from individual mice at day 14 P.I. to assign them to groups (seven/group) as above. Mice were weighed day 15 P.I. and given relevant treatments *per os* as described above for *T* *. muris* . Parasite burdens were determined at day 20 P.I. as per standard protocol [29].

The generalized anthelmintic dose used with each parasite was based on comparisons with previously reported findings [23,26,33–36].

### Data Analyses

Percentage survival and motility scores (averaged) for *in vitro* experiments were combined using code written in MATLAB (R2012a, MathWorks, Massachusetts, U.S.A.) and plotted using Prism 5 (GraphPad Software Inc., La Jolla, CA, U.S.A.). Inhibitory concentration 50% (IC_50_) values were calculated using XLSTAT 2013 (Addinsoft). Worm burdens within each parasite/host system were plotted using Prism 5 and compared using one-way ANOVA and Dunnett’s post-test when more than one drug was involved (Prism 5). When only one drug was to be compared to control, a one-tailed T-test was used.

## Results

### *In vitro* effects of all major anthelmintic classes on representative human STH species

Four clinically-used drugs in all the major classes of anthelmintics were selected for this study-the benzimidazole albendazole (ALB), the nAChR agonist pyrantel (PYR), the macrocyclic lactone ivermectin (IVM), and nitazoxanide (NTZ). These drugs were all tested against adult or L4 stages of one representative species from each of the major human STH parasites-the hookworm parasite *A. ceylanicum* (a significant human parasite in parts of Southeast Asia [37] and closely related to the major hookworm parasite *A* *. duodenale* ), the whipworm parasite *T* *. muris* (closely related to human whipworm *T* *. trichiura* [38]), and *A* *. suum* (very closely related to, if not the same species as, the human parasite *A* *. lumbricoides* [39]). *Heligmosomoides* *bakeri* was used as a non-human parasitic nematode that chronically infects mice and has been used for anthelmintic discovery and development in veterinary medicine [12]. This nematode is in the order Strongylida, which includes important veterinary parasites of ruminants and pigs [40]. We also included a free-living laboratory nematode *C. elegans*, which has also been used or proposed to be used for anthelmintic screening [8,41].

Intoxication was scored using motility as a read-out. Motility is the most common criterion used to evaluate the anthelmintic activity against different nematodes *in vitro* [26,42–44]. Inhibition of parasite motility by drugs is also important clinically because paralysis of STHs *in vivo* by anthelmintics is thought to play an important role in parasite clearance [45]. The motility of each adult was assessed as either + (moved with or without touching) or – (did not move even when touched). To test the utility of collecting more detailed motility information, parasites (and not *C. elegans*) were also scored on a semi-quantitative motility index score (3-0; see Materials and Methods).

### *In vitro* effects of drugs on *A. ceylanicum*

**Figure 1:**
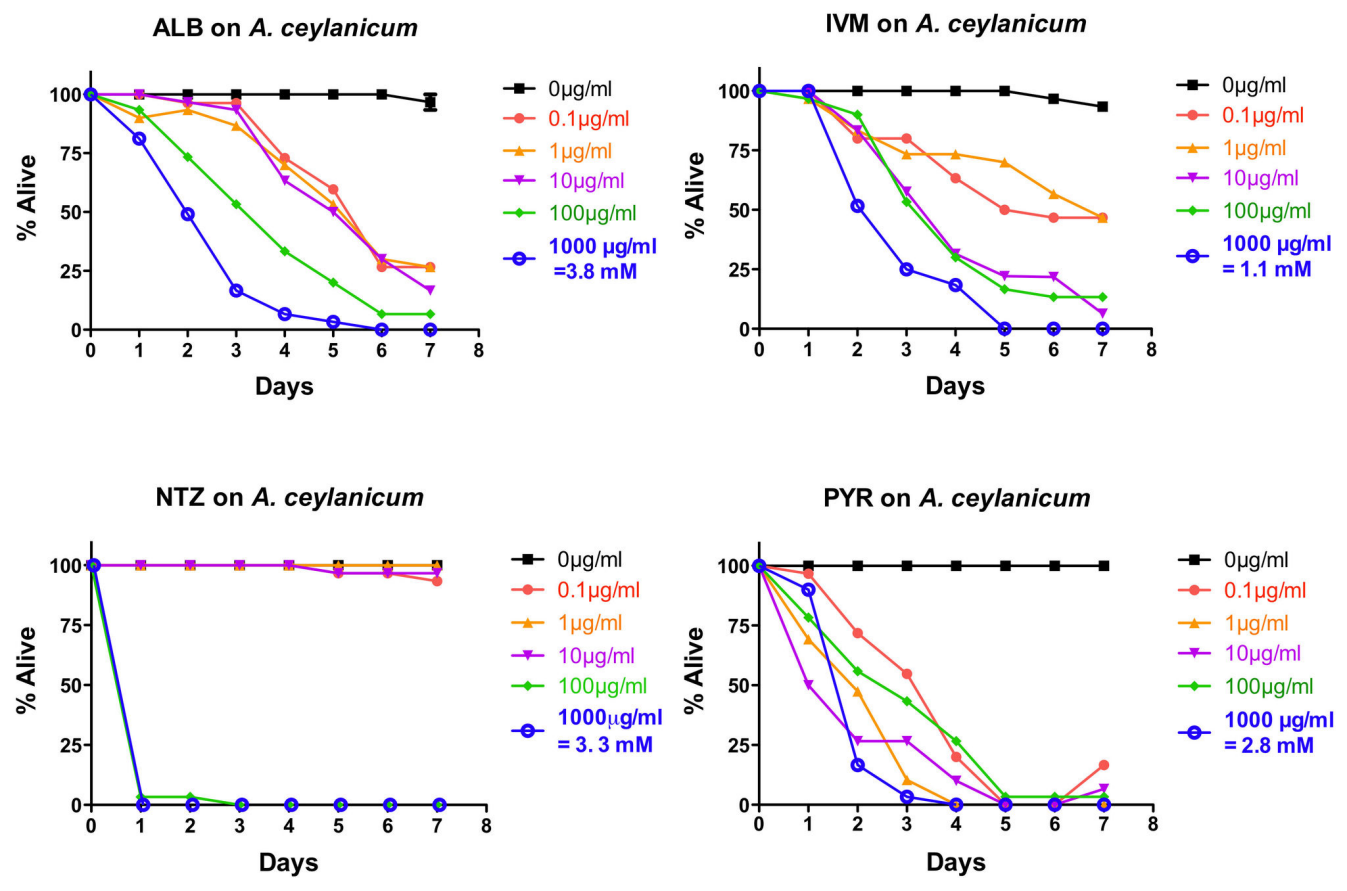
Intoxication of *Ancylostoma ceylanicum* adults *in* *vitro* using five doses of albendazole (ALB), ivermectin (IVM), nitazoxanide (NTZ), or pyrantel (PYR). For comparative reference, the molar concentration of each drug at 1000 µg/mL is indicated. Parasites were scored daily for motility. Any motility, including motility after prodding, was counted as “alive.” Lack of motility, including the lack of a response after prodding, was counted as “dead.” Data are the combination of three independent trials with 10 parasites/trial.

*Ancylostoma ceylanicum* adults were exposed *in vitro* to five doses of each drug: 0.1 µg/mL, 1.0 µg/mL, 10.0 µg/mL, 100 µg/mL, and 1000 µg/mL with ~30 hookworm adults/drug/dose (~10 worms/drug/dose with three independent trials) and examined daily for motility. The results using the +/- score are shown in Figure 1 (combined data from all three experiments). PYR appears to be the generally most potent of the drugs, with quicker intoxication than ALB or IVM at almost all doses and more penetrant intoxication than all the other anthelmintics at the lowest two doses. In general, ALB and IVM show a well-behaved dose–response curve (higher doses generally showing higher intoxication) with full intoxication at the highest doses at around 5-6 days. The dose response with PYR is narrower, e.g., intoxication at 100 µg/mL is markedly lower than might be predicted from the effects at other doses. NTZ shows a very different response, with rapid and complete intoxication at the two highest doses and no effect at any of the other doses.

The semi-quantitative motility index score shows similar overall trends but is more sensitive (Figure S1). Effects with ALB, IVM, and PYR are more rapidly seen with this scoring method and stronger effects at lower doses are seen. A similar all-or-(nearly) nothing response is seen with NTZ in this scoring method.

To get a more quantitative assessment of the *A. ceylanicum* data, we calculated the IC_50_ (concentration at which half the parasites are completely paralyzed) at four days using the +/- scoring data in Figure 1. Four days was chosen as the time point that allowed the best calculation of IC_50_ values for the most drugs. IC_50_ values confirm PYR is the most effective in this assay (Table 1). ALB and IVM are also potent and similar in IC_50_ values (Table 1). NTZ was the least effective (Table 1).

**Table 1:** IC_50_ values (µg/mL) at 4 days.

	ALB	IVM	NTZ	PYR
*A. ceylanicum*	6.46 (2.39-17.00)	2.97 (0.52-11.38)	31.56	0.1<
*A* *. suum*	>1000	>1000	4.69 (3.01-7.41)	25.92 (4.97-230.31)
*T* *. muris*	>1000	>1000	1.63 (1.03-2.71)	63.42 (37.72-109.57)
*H* *. bakeri*	>1000	>1000	6.54 (9.97-10.04)	45
*C. elegans*	>1000	>1000	>1000	746.86 (231-5484.47)

### *In vitro* effects of drugs on *A* *. suum*

**Figure 2:**
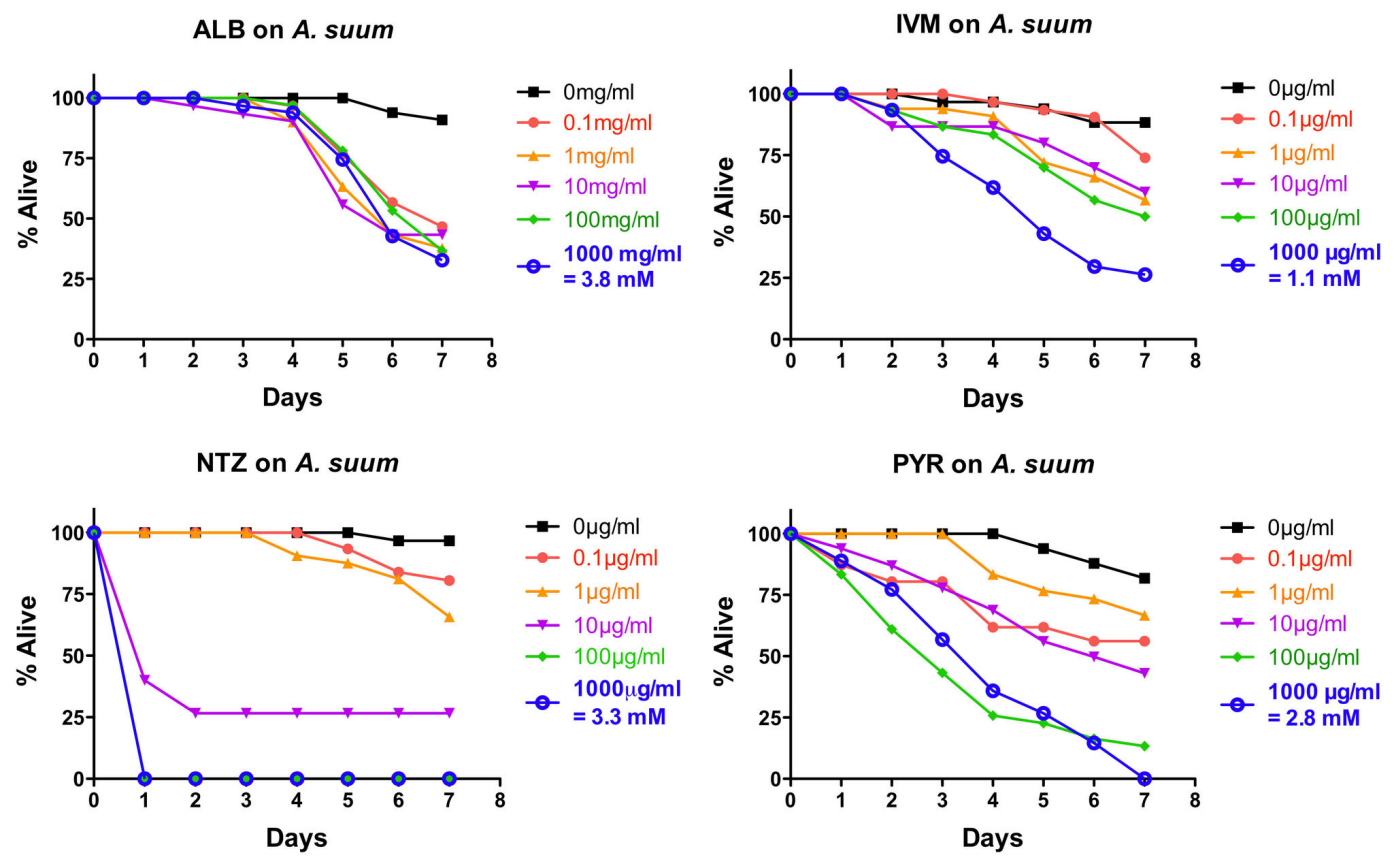
Intoxication of *Ascaris* *suum* fourth-stage larvae (L4) *in* *vitro* using five doses of albendazole (ALB), ivermectin (IVM), nitazoxanide (NTZ), or pyrantel (PYR). For comparative reference, the molar concentration of each drug at 1000 µg/mL is indicated. Parasites were scored daily for motility. Any motility, including motility after prodding, was counted as “alive.” Lack of motility, including lack of a response after prodding, was counted as “dead.” Data are the combination of three independent trials with 10 parasites/trial.

*Ascaris* *suum* L4 were isolated from pig intestines on day 14 P.I. and exposed *in vitro* to five doses of each drug as per *A. ceylanicum* adults (0.1 µg/mL, 1.0 µg/mL, 10.0 µg/mL, 100 µg/mL, and 1000 µg/mL) with ~30 *A* *. suum* L4 /drug/dose (~10 worms/drug/dose with three independent repeats). These assays were examined daily for motility. The results using the +/- score are shown in Figure 2 (combined data from all three experiments). As with *A. ceylanicum*, NTZ showed a steep response, with strong intoxication at higher doses and weak intoxication at lower doses. PYR was much more intoxicating than ALB or IVM at the two highest doses. ALB was better than IVM at all but the highest dose and was better than PYR at the three lowest doses. The response to ALB was similar over four logarithms of doses (i.e., no strong dose–response). With ALB, IVM, and PYR at all doses, the level of intoxication with *A* *. suum* was noticeably weaker (in terms of penetrance and rapidity of intoxication) and than with *A. ceylanicum*.

The motility index score for each drug is shown in Figure S2. The relative conclusions are similar as with the +/- score except that effects with ALB, IVM, and PYR are more rapidly detected in this scoring system. This rapidity is most pronounced with IVM, which shows a very rapid intoxication at even the lowest doses. These data indicate that the drugs are more capable of reducing motility (Figure S2) than causing complete paralysis (Figure 2; which counts as motile a worm that moves if untouched or touched). If one assumes a worm that does not move upon touching is dead, then these data indicate the drugs are better at reducing *A* *. suum* larval motility than killing them. A similar conclusion is evident for *A. ceylanicum*. For NTZ, on the other hand, a comparison of Figure S2 and Figure 2 is consistent with an all-or-nothing effect of NTZ on the parasite (the intoxication seen at 0.1 µg/mL and 1 µg/mL in Figure S2 parallels that of DMSO controls and does not appear to be significant).

As with *A. ceylanicum*, IC_50_ values for each drug using the +/- scoring data at four days were calculated for *A* *. suum* larvae (Table 1). These values confirm the weaker effects of ALB, IVM, and PYR on *A* *. suum* than on *A. ceylanicum*.

### *In vitro* effects of drugs on *T* *. muris*

**Figure 3:**
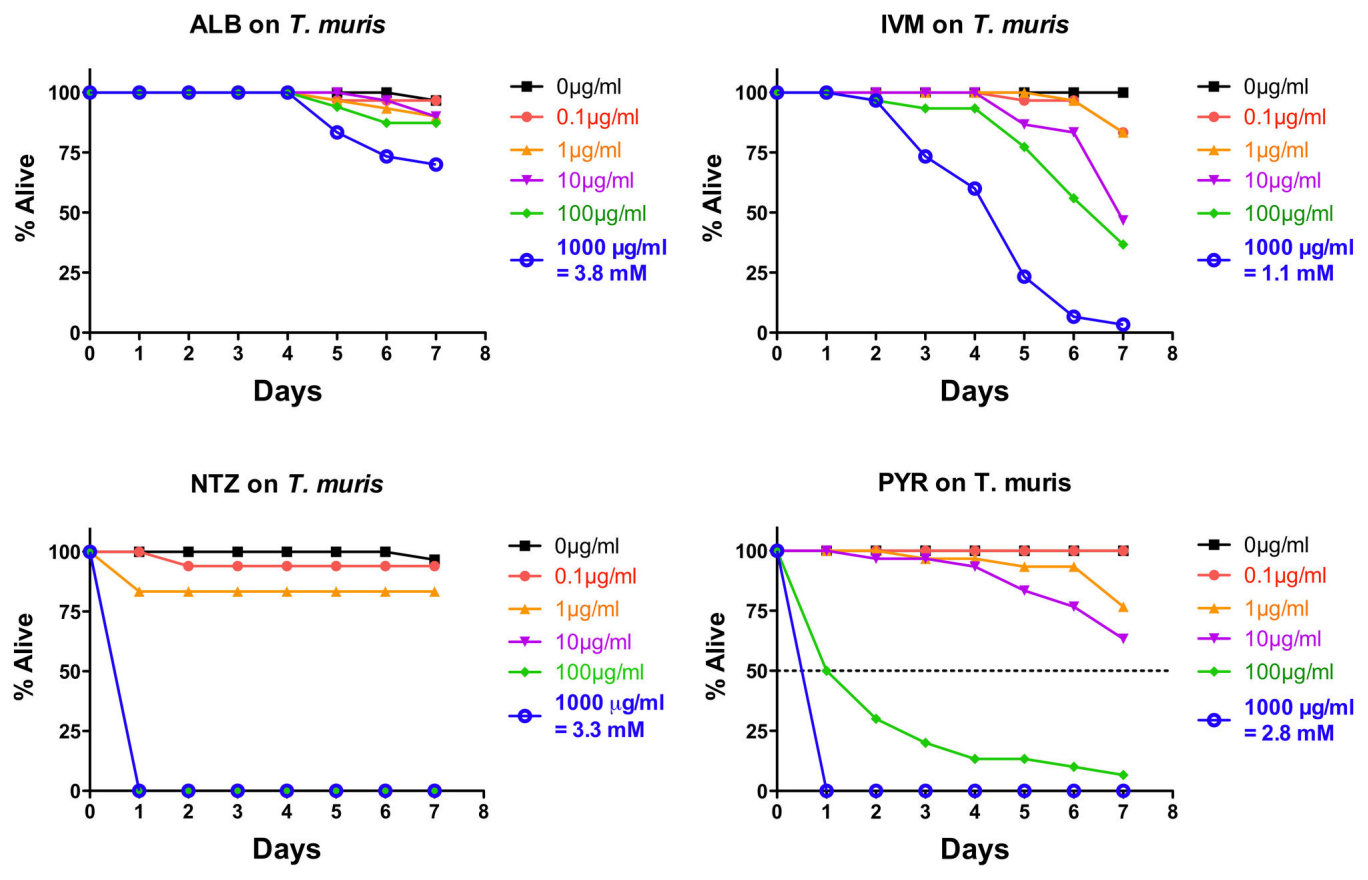
Intoxication of *Trichuris* *muris* adults *in* *vitro* using five doses of albendazole (ALB), ivermectin (IVM), nitazoxanide (NTZ), or pyrantel (PYR). For comparative reference, the molar concentration of each drug at 1000 µg/mL is indicated. Parasites were scored daily for motility. Any motility, including motility after prodding, was counted as “alive.” Lack of motility, including lack of a response after prodding, was counted as “dead.” Data are the combination of three independent trials with 10 parasites/trial.

*Trichuris* *muris* whipworm adults were treated with ALB, IVM, NTZ, and PYR at the same doses as above, and monitored daily for motility using the +/- score (Figure 3; ~30 adults/drug/dose; data combined from three independent experiments). Both NTZ and PYR show a steep dose–response with rapid and penetrant intoxication at the highest doses and little/no intoxication at the lower doses. IVM shows a weaker effect than NTZ and PYR at the higher doses; none show a strong effect at lower doses (Figure 3). IVM is nonetheless somewhat effective at higher doses and at later time points. Surprisingly, ALB has very little or no effect at all the doses tested. The response to ALB in *T* *. muris* is a marked contrast to the previous two parasites.

Assessing the parasites with the motility index score gives a similar impression (Figure S3). With NTZ and PYR, a rapid, strong intoxication is seen at higher doses (Figure S3). Effects at lower doses parallel those seen in DMSO only control, suggesting no significant effect at these doses (Figure S3). The effects of IVM at the higher doses are more pronounced with this motility index scoring system (Figure S3) suggesting that, relative to other anthelmintics, IVM is more effective at reducing motility of parasites than it is at completely immobilizing or killing them. At all doses, ALB is only slightly better than the DMSO control on motility of *T* *. muris* ; which is consistent with a weak to moderate effect (Figure S3).

Analysis of IC_50_ data using the +/- scoring data at four days with *T* *. muris* confirms these qualitative conclusions (Table 1). Both PYR and NTZ show measurable IC_50_s but ALB or IVM do not (Table 1). With the exception of NTZ, the effects seen are weaker than those seen with *A. ceylanicum* (Table 1).

### *In vitro* effects of drugs on *H* *. bakeri*

**Figure 4:**
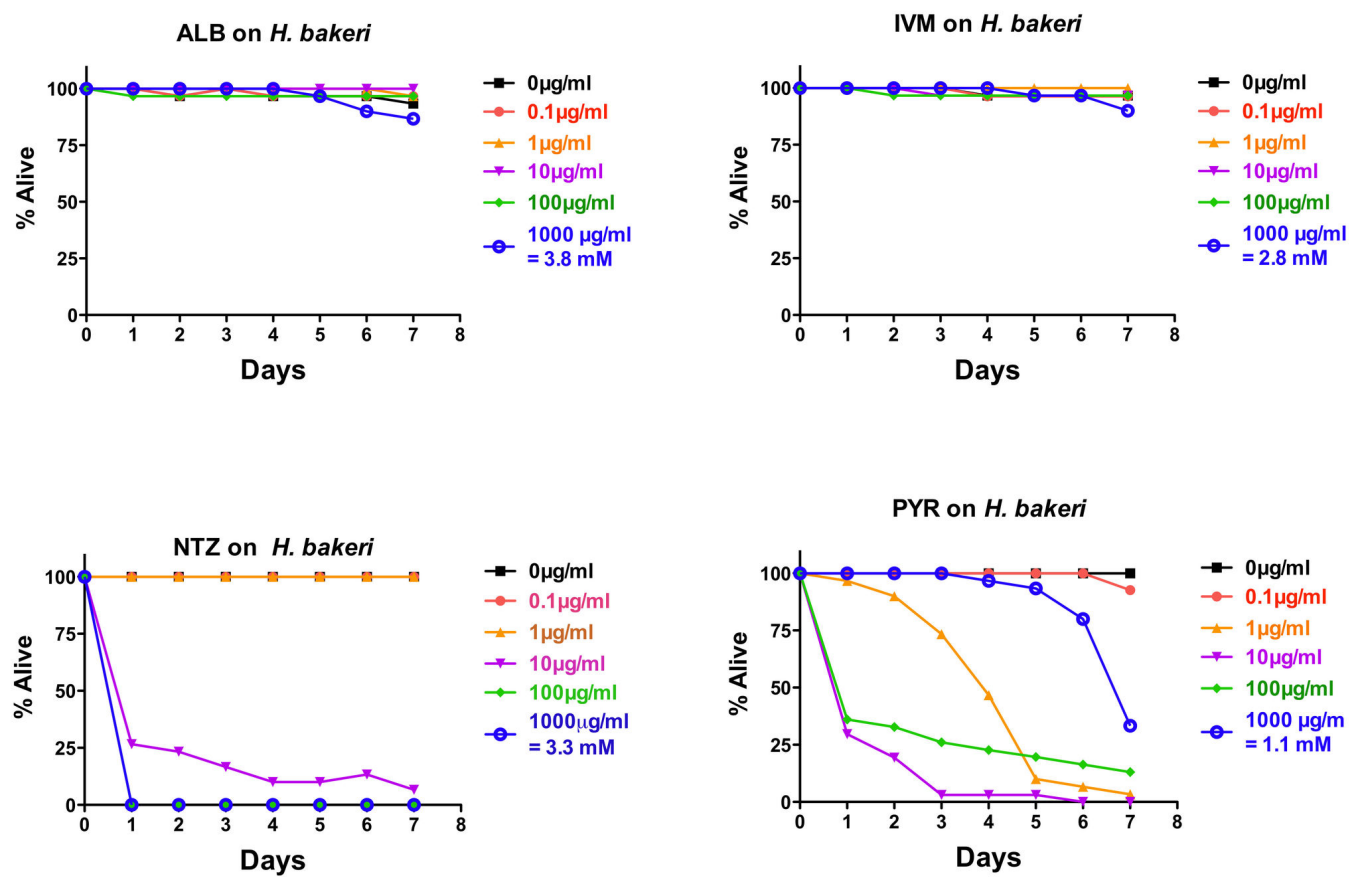
Intoxication of *Heligmosomoides* *bakeri* adults *in* *vitro* using five doses of albendazole (ALB), ivermectin (IVM), nitazoxanide (NTZ), or pyrantel (PYR). For comparative reference, the molar concentration of each drug at 1000 µg/mL is indicated. Parasites were scored daily for motility. Any motility, including motility after prodding, was counted as “alive.” Lack of motility, including a lack of a response after prodding, was counted as “dead.” Data are the combination of three independent trials with 10 parasites/trial.

*Heligmosomoides* *bakeri* was included in this study as an intestinal roundworm that has no obvious relationship to human STHs. Although sometimes called mouse “hookworm,” this parasite is not a hookworm as it does not ingest blood like human hookworms [46]. Adult *H* *. bakeri* parasites were subjected *in vitro* to ALB, IVM, NTZ, and PYR at the same concentrations as above (~30 adult parasites/drug/dose). As with the other parasites using the +/- score, NTZ showed an all-or-nothing type response—rapid and complete or near complete intoxication at high doses and no intoxication at lower doses (Figure 4). Neither ALB nor IVM showed any significant effect (Figure 4). PYR did have a pronounced effect on *H* *. bakeri* , with more penetrant effects at moderate doses and weaker effects at the highest and lowest doses (Figure 4). Thus, PYR showed a U-shaped or bell-shaped response on *H* *. bakeri* , which has been reported before for this drug [47].

Using the motility index score, NTZ and PYR similarly show an all-or-nothing or bell-shaped response respectively (Figure S4). With ALB, IVM, and PYR on *H* *. bakeri* , stronger effects with this scoring system can be ascertained than with the +/- score (Figure S4); although the difference between the two scoring systems is more dramatic for IVM. ALB shows a weaker effect relative to DMSO controls than the other drugs, and, as with *A* *. suum* and *T* *. muris* , no dose–response is seen with this drug (Figure S4).

Analysis of IC_50_ data at four days with *H* *. bakeri* confirmed these qualitative conclusions (Table 1). Both PYR and NTZ show measurable IC50s but not ALB or IVM (Table 1). The lack of 95% confidence interval with PYR is reflective of the U-shaped response.

### Effects of drugs on *C. elegans*

**Figure 5:**
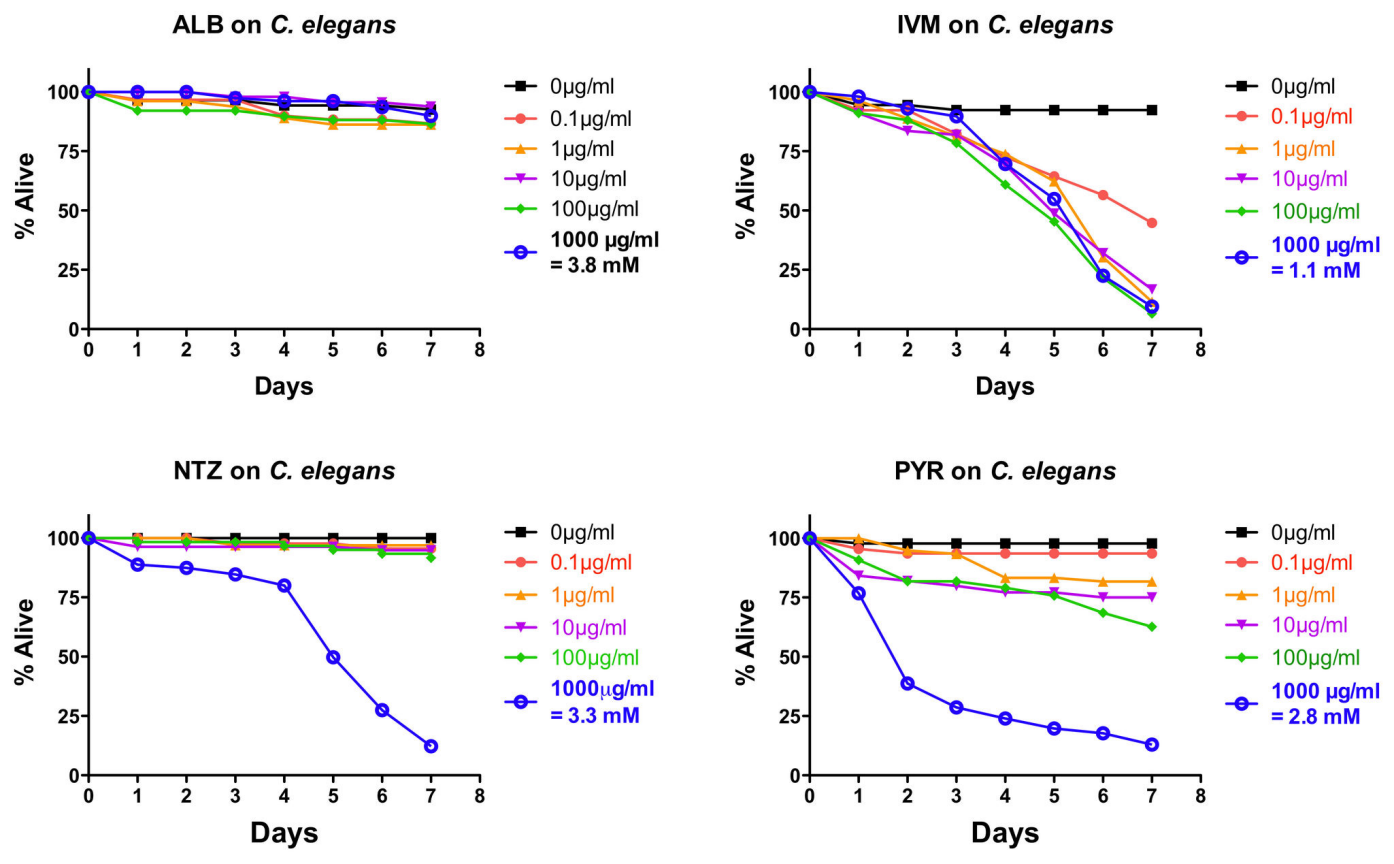
Intoxication of *Caenorhabditis elegans* free-living fourth-stage larvae (L4) using five doses of albendazole (ALB), ivermectin (IVM), nitazoxanide (NTZ), or pyrantel (PYR). For comparative reference, the molar concentration of each drug at 1000 µg/mL is indicated. Roundworms were scored daily for motility. Any motility, including motility after prodding, was counted as “alive.” Lack of motility, including a lack of a response after prodding, was counted as “dead.” Data are the combination of three independent trials with ~10 nematodes/trial.

Since the free-living roundworm *C. elegans* is sometimes used as a readily available laboratory model for studying anthelmintics [8,41], we included it in our study here. *Caenorhabditis elegans* L4-staged were subjected to the same doses of ALB, IVM, NTZ, and PYR in liquid medium (~45 nematodes/drug/dose). With the exception of IVM, the drugs work less well in *C. elegans* than in the parasites as assessed with the +/- score (Figure 5). This result is consistent with previous reports, and likely a consequence of the relative impermeability of the *C. elegans* cuticle [48]. As with other nematodes, NTZ shows an all-or-nothing type response but intoxication is seen only at the highest dose and takes longer (Figure 5). PYR is effective only at the highest dose (Figure 5), and ALB does not show an obvious effect in the +/- scoring system (Figure 5). *Caenorhabditis elegans* is sensitive to IVM at all doses tested, although intoxication as defined by +/- score is not rapid (Figure 5). Of all the drugs, only PYR was able to achieve an IC_50_ value by four days (Table 1).

### *In vivo* single dose studies

**Figure 6:**
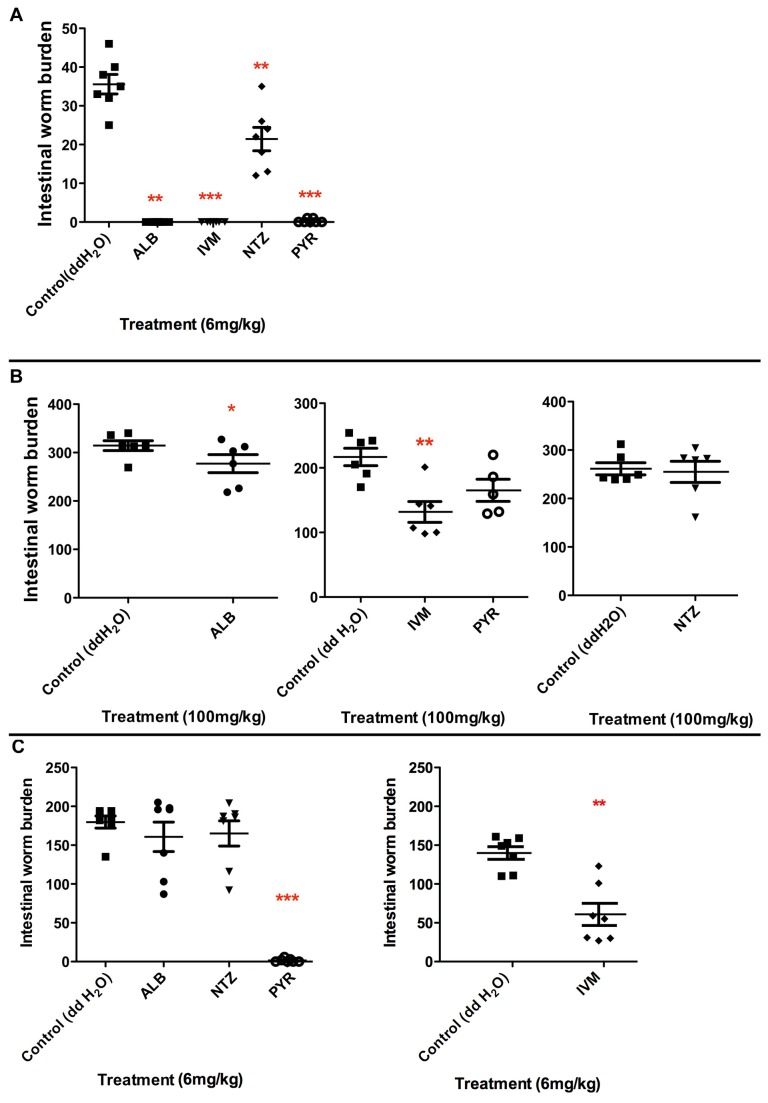
*In* *vivo* efficacies of albendazole (ALB), ivermectin (IVM), nitazoxanide (NTZ), and pyrantel (PYR) against A) *A. ceylanicum* infections in hamsters; B) *T* *. muris* infections in STAT6-/- mice; and C) *H* *. bakeri* infections in Swiss Webster mice. Shown are the intestinal worm burdens after treatment *per os* with the drugs in comparison to placebo (water-only) control. Worm burdens shown in separate panels for the same parasite are from separate experiments; worm burdens shown in the same panel for the same parasite are from a single experiment. P values relative to placebo control are as follows: * < 0.05; ** < 0.01; *** < 0.001.

Since follow up work to *in vitro* studies invariably involve *in vivo* studies, we performed *in vivo* assays with single doses of all four drugs on the parasites (namely *A. ceylanicum*, *T* *. muris* , and *H* *. bakeri* ) that we maintain in rodents. Consistent with our *in vitro* findings, we find that *A. ceylanicum* has the best sensitivity to these drugs *in vivo*, especially with regards to ALB (Figure 6).

## Discussion

We compare *in vitro* for the first time all major anthelmintic classes against nematode species related to all major STHs that infect humans as well as an intestinal parasitic nematode not closely related to those in humans and a free-living nematode. Based on these data, several trends emerge. First, the most sensitive of all the nematodes tested is the hookworm *A. ceylanicum*. In general, the strongest effects with ALB, IVM, and PYR were found with this parasite (Table 1). Indeed, it was the only nematode for which the IC_50_s of ALB and IVM could be calculated at four days. The second most sensitive was *A* *. suum* . With the exception of IVM and the bell-shape response with PYR on *H* *. bakeri* , *T* *. muris* and *H* *. bakeri* had broadly similar responses to the anthelmintics tested, while *C. elegans* had the weakest response. We note that the most severe phenotype we scored was the failure of the nematodes to move when prodded (i.e., completely paralyzed). Although we cannot be certain that such nematodes were dead (since we did not test what would happen if we allowed the nematodes to recover in the absence of drug after the experiments were complete on day seven), the complete paralysis of nematodes, whether dead or not, is a powerful phenotype and a standard in the field of anthelmintic studies [26,42–44].

Certain other trends are also evident. NTZ shows a steep all-or-nothing response with all nematodes tested. Across the parasites, the other drugs (ALB, IVM, PYR) show a preferential ability to cause partial paralysis over complete paralysis/death. By comparing the +/- score with the motility index score, one can see that this effect is most pronounced for IVM. This result might explain why IVM is not as useful as ALB or PYR for STH chemotherapy, in particular against hookworms that have their heads buried in the intestinal mucosa and are therefore more difficult to dislodge by partial paralysis. Comparison of various scoring methods can thus be useful to ascertain certain aspects of the effects of anthelmintics.

For general human chemotherapy against STHs, ALB is superior to all the other anthelmintics tested here and is considered the single best anthelmintic. Given that its effects were clearly most readily seen with *A. ceylanicum* and given the overall sensitivity of this parasite to all the anthelmintics *in vitro*, then our data indicate that of all the nematodes tested, this parasite is a better choice to include early on in drug screening for broad-spectrum human STH chemotherapy (e.g., of all the nematodes tested here, it would have the greatest chance of discovering ALB in a drug screen). In principle, one would want to include as many parasites as possible, but this is not always practical or cost-effective. Furthermore, the good sensitivity of *A. ceylanicum in vitro* to drugs is also reflected in its good sensitivity *in vivo* to drugs, including, most importantly, to ALB. This finding suggests *A. ceylanicum* is also a good initial choice for *in vivo* studies that follow up from *in vitro* experiments. Another advantage of using a sensitive nematode like *A. ceylanicum* for drug screening is that one can potentially find families of active compounds that could be optimized by screening with expanded repertoires of compounds with similar scaffolds. Thus, our data indicate that the use of *A. ceylanicum* early on in the screening process will help identify compounds particularly useful for broad human STH chemotherapy.

Notably, our data indicate that choosing a specific parasite for *in vitro* screening for drugs against that parasite, although intuitively correct, does not always give the best results. For example, NTZ is perhaps more potent on *T* *. muris* than on any other parasite and yet is a poor choice for single-dose whipworm chemotherapy in humans [49]. Similarly, our *in vitro* results suggest that PYR is better than ALB in its effects against *T* *. muris* but this finding is not reflected in clinical findings where the two drugs are similar [6]. Thus, screening on *T* *. muris* *in vitro* itself will not always yield the best clinical advice for drugs treating this parasite. This result, of course, may be a consequence of *in vivo* pharmacokinetics, metabolism, and interaction with the host because of intraepithelial cell location of this parasite in the large intestine. This is a limitation of *in vitro* assays.

Interestingly, our results suggest that NTZ will only be effective against any parasite when a certain threshold is reached. Our finding may explain why the most effective therapy with NTZ involves multiple doses over multiple days, perhaps what is required to achieve and maintain such a threshold. Our study suggests that pharmakinetic studies with NTZ in animal models are important for determining the optimal strategy for use with this drug.

PYR is another drug with interesting effects. In general, it is the most potent of all the drugs *in vitro* (except for NTZ). Invariably, however, the dose response with this drug is not straightforward. For example, with *A. ceylanicum* the 100 µg/mL dose is misplaced; with *A* *. suum* the 0.1 µg/mL dose is misplaced, and with *H* *. bakeri* a bell-shaped response is seen. These results suggest that careful dosing with PYR may be required to achieve the best clinical outcome and may actually differ from parasite to parasite. Thus, the optimal clinical effects with PYR may not have yet been reached.

The data with *A* *. suum* collected here differ from the other parasites in two ways. The *in vitro* studies, although carried out with intestinal staged parasites, involve larvae and not adults. The reason is practical—it is very difficult to collect enough adult staged parasites from pigs infected under controlled conditions for these studies (see Materials and Methods; adult parasites recovered from farm pigs processed at an abattoir are extremely variable and do not possess the quality required for critical assessment of *in vitro* anthelmintic efficacy here). In addition, *in vivo* efficacy of the four anthelmintics on *Ascaris* infections in pigs was not carried out for reasons of practicality and cost. Nonetheless, based on available data, *A* *. suum* infections in pigs may not have superior sensitivity to *A. ceylanicum* infections in hamsters for ALB or PYR (cleared with 6 mg/kg dose in this study) where the recommended doses in pigs are 5-10 mg/kg and 22 mg/kg respectively.

In summary, our data indicate that comparison of the effects of drugs on a variety of parasitic nematodes *in vitro* can yield information that might inform use of these drugs clinically. Our data, for example, suggest that maximizing the clinical value of various anthelmintics could be done by altering the treatment routine and/or dosing. Our data also indicate that the clinical value of a drug cannot always be predicted by *in vitro* assays, suggesting that follow up to *in vitro* screening should include *in vivo* pharmacokinetics/metabolic studies. Whereas drug screening should undoubtedly be done in the particular human parasite of interest, using *A. ceylanicum* to finding a broad-spectrum anthelmintics for human therapy is a promising and efficient starting point because of its sensitivity to drugs and its relative response to drugs that have the best clinical value.

## Supporting Information

Figure S1 Intoxication of *Ancylostoma ceylanicum* adults *in vitro* using five doses of either albendazole (ALB), ivermectin (IVM), nitazoxanide (NTZ), or pyrantel (PYR) as scored by motility index. Parasites were scored daily for motility on a scale of 3-0. Data are from the same experiments in Figure 1.
(JPG)

Figure S2 Intoxication of *Ascaris* *suum* fourth-stage larvae (L4) *in vitro* using five doses of either albendazole (ALB), ivermectin (IVM), nitazoxanide (NTZ), or pyrantel (PYR). Parasites were scored daily for motility on a scale of 3-0. Data are from the same experiments in Figure 2.
(JPG)

Figure S3 Intoxication of *Trichuris* *muris* adults *in vitro* using five doses of either albendazole (ALB), ivermectin (IVM), nitazoxanide (NTZ), or pyrantel (PYR). Parasites were scored daily for motility on a scale of 3-0. Data are from the same experiments in Figure 3.
(JPG)

Figure S4 Intoxication of *Heligmosomoides* *bakeri* adults *in vitro* using five doses of either albendazole (ALB), ivermectin (IVM), nitazoxanide (NTZ), or pyrantel (PYR). Parasites were scored daily for motility on a scale of 3-0. Data are from the same experiments in Figure 4.
(JPG)
