# Supplementary figures and images for: An Extensive Comparison of the Effect of Anthelmintic Classes on Diverse Nematodes
Source: PLoS One. 2013 Jul 15;8(7):e70702. doi: 10.1371/journal.pone.0070702 (PMC3712009; doi:10.1371/journal.pone.0070702)

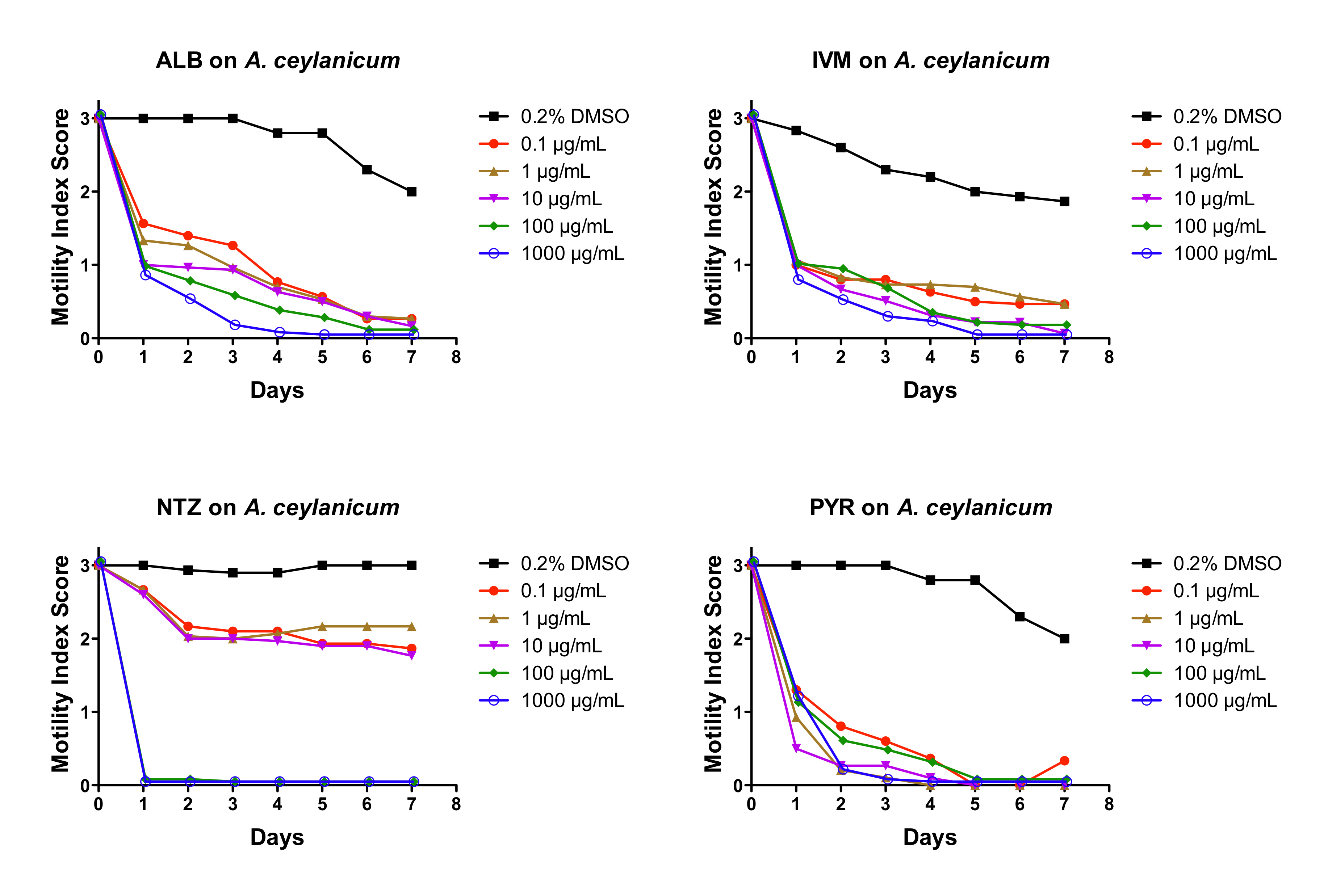

Supplement: Figure S1 — Parasites were scored daily for motility on a scale of 3-0. Data are from the same experiments in Figure 1. (JPG) [file pone.0070702.s001.jpg]

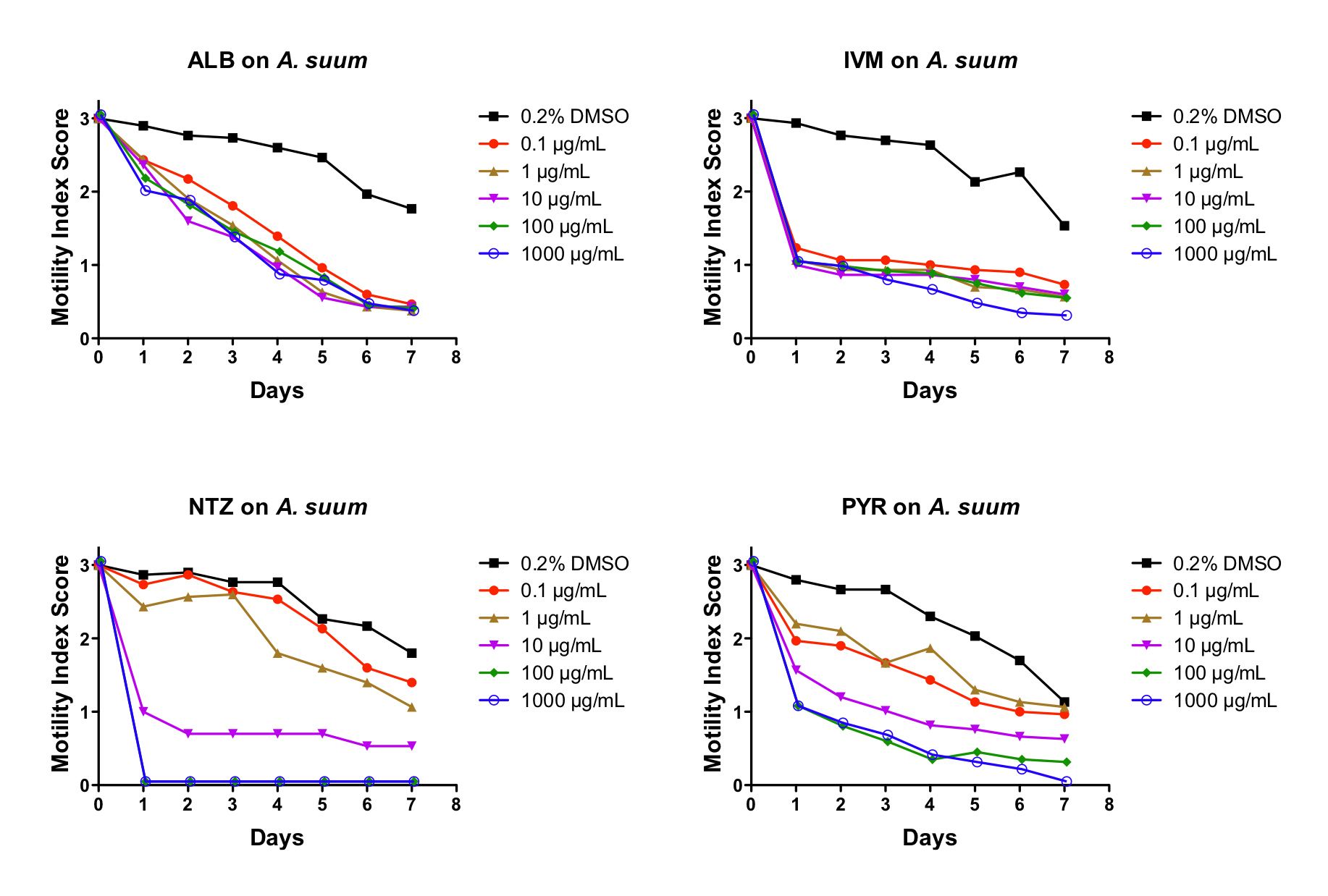

Supplement: Figure S2 — Parasites were scored daily for motility on a scale of 3-0. Data are from the same experiments in Figure 2. (JPG) [file pone.0070702.s002.jpg]

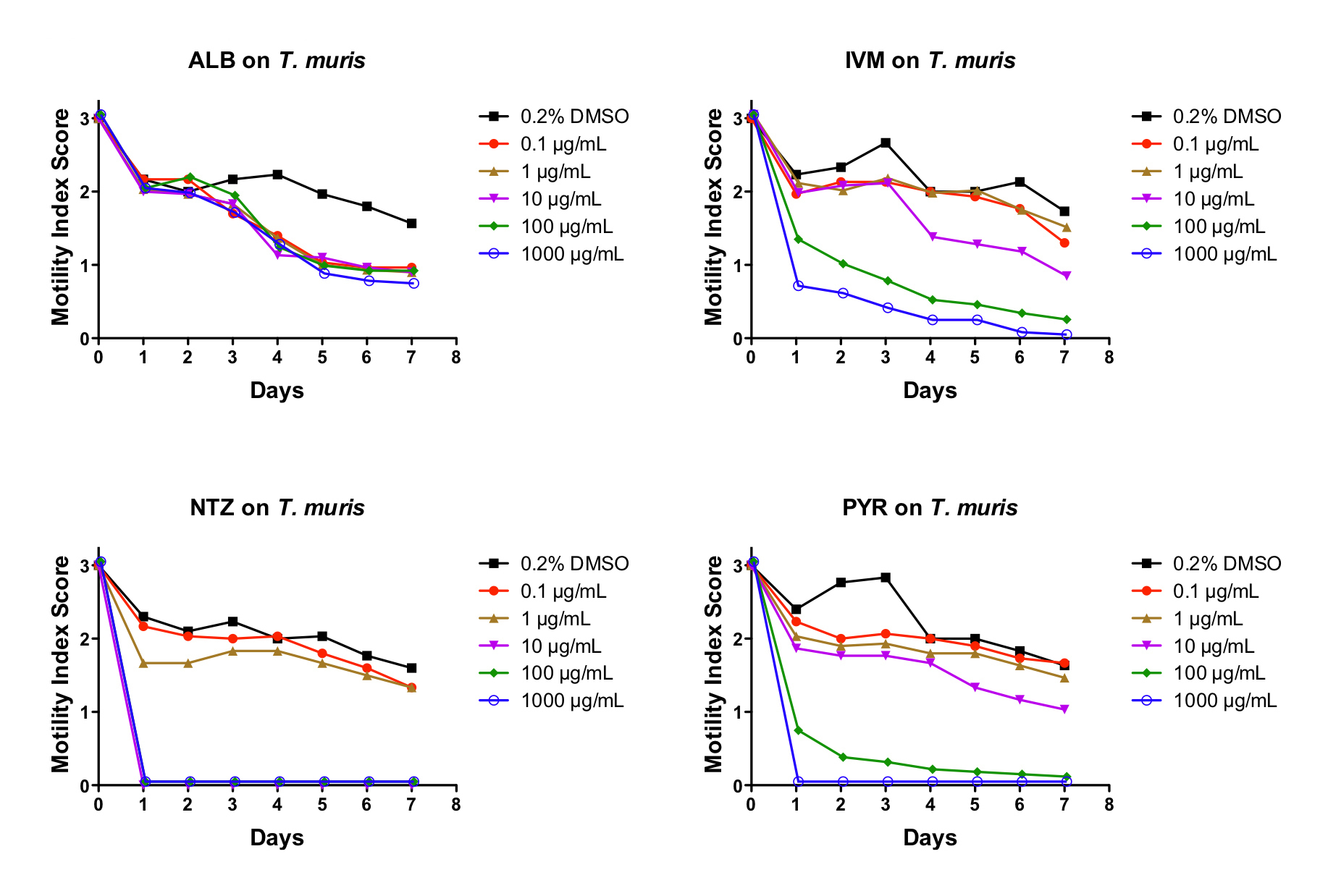

Supplement: Figure S3 — Parasites were scored daily for motility on a scale of 3-0. Data are from the same experiments in Figure 3. (JPG) [file pone.0070702.s003.jpg]

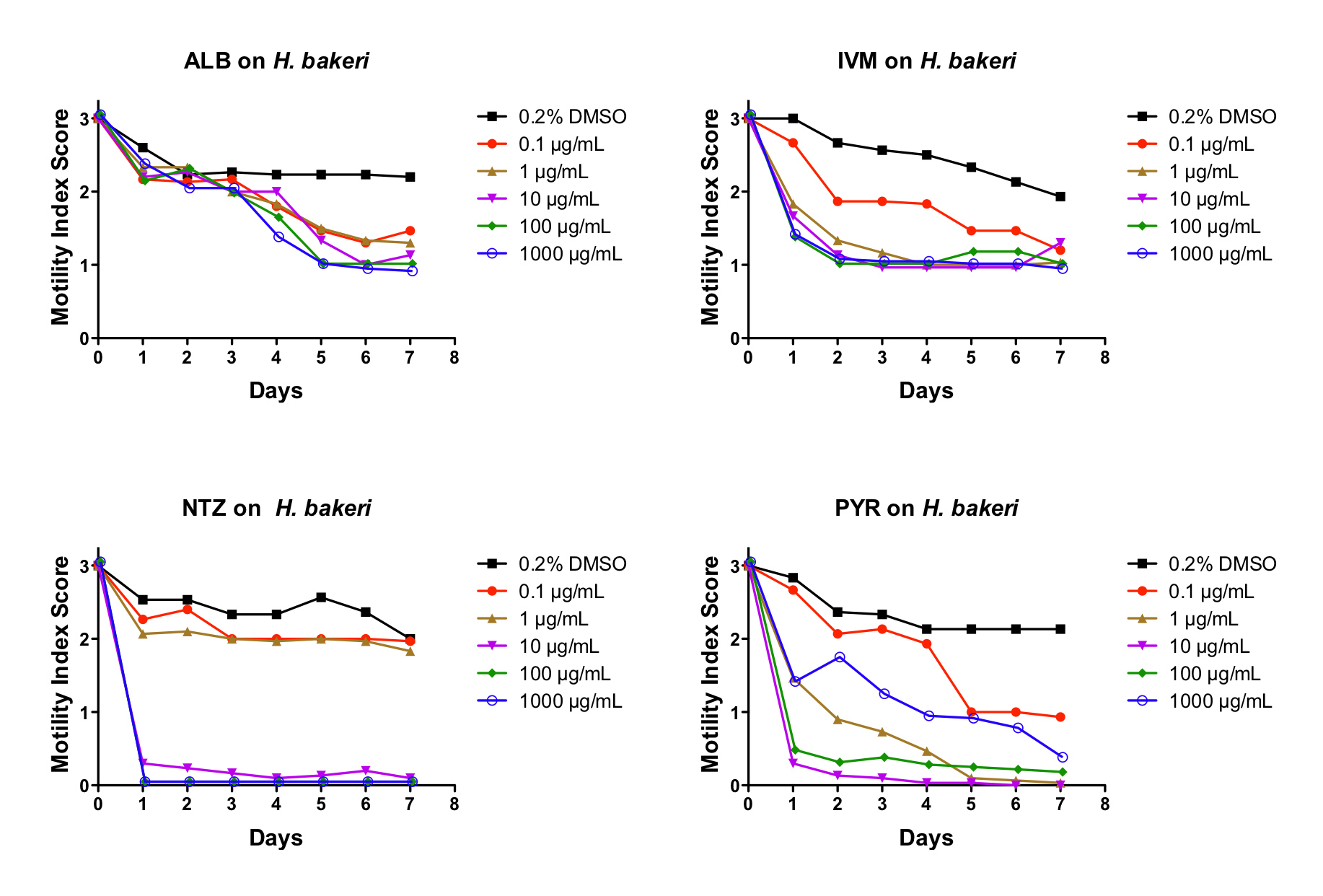

Supplement: Figure S4 — Parasites were scored daily for motility on a scale of 3-0. Data are from the same experiments in Figure 4. (JPG) [file pone.0070702.s004.jpg]
